# Reply to Cheong and Jones: The role of science in responding to collective behavioral threats

**DOI:** 10.1073/pnas.2114477118

**Published:** 2021-10-15

**Authors:** Joseph B. Bak-Coleman, Carl T. Bergstrom

**Affiliations:** ^a^Center for an Informed Public, University of Washington, Seattle, WA 98195;; ^b^eScience Institute, University of Washington, Seattle, WA 98195;; ^c^Department of Biology, University of Washington, Seattle, WA 98195

In our PNAS article “Stewardship of global collective behavior” (1), we describe the breakneck pace of recent innovations in information technology. This radical transformation has transpired not through a stewarded effort to improve information quality or to further human well-being. Rather, current technologies have been developed and deployed largely for the orthogonal purpose of keeping people engaged online. We cannot expect that an information ecology organized around ad sales will promote sustainability, equity, or global health. In the face of such impediments to rational democratic action, how can we hope to overcome threats such as global warming, habitat destruction, mass extinction, war, food security, and pandemic disease? We call for a concerted transdisciplinary response, analogous to other crisis disciplines such as conservation ecology and climate science.

In their letter (2), Cheong and Jones share our vision of the problem—but they express frustration at the absence of an immediately actionable solution to the enormity of challenges that we describe. They assert “swarm intelligence begins now or never” and advocate local, authentic, and immediate “scale reduction.” It’s an appealing thought: Let us counter pathologies of scale by somehow reversing course.

But it’s not clear what this would entail by way of practical, safe, ethical, and effective intervention. Have there ever been successful, voluntary, large-scale reductions in the scale of any aspect of human social life?

Nor is there reason to believe that an arbitrary, hasty, and heuristically decided large-scale restructuring of our social networks would reduce the long tail of existential risk. Rather, rapid shocks to complex systems are a canonical source of cascading failure (3). Moving fast and breaking things got us here. We can’t expect it to get us out.

Nor do we share the authors’ optimism about what scientists can accomplish with “a collective chorus … through every digital channel” (2). It is difficult to envision a louder, more vehement, and more cohesive scientific response than that to the COVID-19 pandemic. Yet this unified call for basic public health measures—grounded in centuries of scientific knowledge—nonetheless failed to mobilize political leadership and popular opinion.

Our views do align when it comes to the “now-or-never urgency” that Cheong and Jones (2) highlight. Indeed, this is a key feature of a crisis discipline: We must act without delay to steer a complex system—while still lacking a complete understanding of how that system operates (4).

As scholars, our job is to call attention to underappreciated threats and to provide the knowledge base for informed decision-making. Academics do not—and should not—engage in large-scale social engineering. Our grounded view of what science can and should do in a crisis must not be mistaken for lassitude or unconcern. Worldwide, the unprecedented restructuring of human communication is having an enormous impact on issues of social choice, often to our detriment. Our paper is intended to raise the alarm. Providing the definitive solution will be a task for a much broader community of scientists, policy makers, technologists, ethicists, and other voices from around the globe.

## References

[1] J. B. Bak-Coleman ., Stewardship of global collective behavior. Proc. Natl. Acad. Sci. U.S.A. 118, e2025764118 (2021). 34155097 10.1073/pnas.2025764118 PMC8271675

[2] K. H. Cheong, M. C. Jones, Swarm intelligence begins now or never. Proc. Natl. Acad. Sci. U.S.A., 10.1073/pnas.2113678118 (2021). PMC8545451 34654748

[3] M. Scheffer ., Anticipating critical transitions. Science 338, 344–348 (2012). 23087241 10.1126/science.1225244

[4] M. E. Soulé, What is conservation biology? Bioscience 35, 727–734 (1985).

